# Metagenomic sequencing for identification of nontuberculous mycobacteria and other pathogens in patients with mixed infection of the lung

**DOI:** 10.3389/fcimb.2025.1592216

**Published:** 2025-06-19

**Authors:** Jinyan Yu, Xuejiao Lv, Qi Wang, Mingyue Gao, Wei Li

**Affiliations:** ^1^ Department of Respiratory and Critical Care Medicine, The Second Hospital of Jilin University, Changchun, Jilin, China; ^2^ Department of Nuclear Medicine, The Second Hospital of Jilin University, Changchun, Jilin, China

**Keywords:** non-tuberculous mycobacteria (NTM), metagenomic next generation sequencing (mNGS), mixed infection (MI), bronchoalveolar lavage fluid (BALF), diagnosis, treatment

## Abstract

**Background:**

It can be difficult to distinguish lung disease caused by nontuberculous mycobacteria (NTM), *Mycobacterium tuberculosis*, and mixed infections (MIs) that include NTM. Metagenomic next generation sequencing (mNGS) is a highly sensitive method that can reliably identify lung pathogens. We retrospectively analyzed the records of patients who had MIs of the lungs that included NTM and received mNGS testing.

**Methods:**

The records of 36 patients who were diagnosed with NTM infection of the lungs at the Second Hospital of Jilin University from Nov 2023 to Jun 2024 were analyzed. Initial empirical treatments were ineffective in all patients, leading to the application of mNGS of bronchoalveolar lavage fluid (BALF).

**Results:**

The average patient age was 62.4 years, 22 patients had one or more underlying chronic disease, and all patients had at least one respiratory symptom (cough, sputum production, fever, or dyspnea). Chest CT examinations showed that patients had different degrees of pneumonia and pleural effusion. Among tested patients, there were elevated levels of erythrocyte sedimentation rate in 81.8% (18/22) and elevated C-reactive protein in 90.5% (19/21). There were variable results from acid-fast staining of bronchoalveolar lavage fluid (BALF; 3/36, 8.3%), and transbronchial lung biopsy (TBLB; 5/14, 35.7%). mNGS identified seven NTM species. Treatment based on the mNGS results led to the resolution of clinical symptoms and absorption of lung lesions in all patients.

**Conclusions:**

Most of the 36 patients with MIs of the lungs that included NTM had underlying diseases. The results of traditional tests, including sputum or BALF culture and smear, acute phase markers, and TBLB pathological examination, were problematic. mNGS provides rapid and reliable diagnosis, allowing the rapid implementation of appropriate therapy in patients with MIs of the lungs that include NTM.

## Introduction

Non-tuberculous mycobacteria (NTM) pulmonary diseases are systemic lung infections that can threaten human life and well-being, and are an increasingly serious global health problem. Recent changes in the global climate and the development of more sophisticated technologies for identification of pathogens have led to the increased detection of NTM and other pathogens (Porvaznik et al., 2017; Chin et al., 2020; Gopalaswamy et al., 2020; Sharma and Upadhyay, 2020).

Identification of the etiological pathogen is essential for the clinical diagnosis of NTM lung disease. However, the traditional culture methods have low rates of positive identification and are time-consuming, making them unsuitable for obtaining rapid and accurate clinical diagnoses. It is also difficult to distinguish between infections by NTM and by *Mycobacterium tuberculosis*, and these infections require different treatments (Kalaiarasan et al., 2020). For a patient who has a mixed infection (MI) with an NTM, the signs of the NTM infection may be masked by a strong pathogenic response to the more easily detected co-infecting pathogen, resulting in persistent symptoms, prolonged treatment time, increased use of medical resources, and poor prognosis (Cullen et al., 2000). The limitations of traditional methods are especially problematic for patients with atypical clinical symptoms and imaging results, and for patients who have multiple negative sputum smears and negative culture results for acid-fast bacilli. Reliance on the traditional methods for pathogen identification often leads to misdiagnosis and inappropriate treatment (Quan et al., 2017; Kalaiarasan et al., 2020; Morais et al., 2022; Khare and Brown-Elliott, 2023). Metagenomic next generation sequencing (mNGS) can identify and type all pathogens in a clinical sample by directly measuring DNA and RNA (Zheng et al., 2021; Hilt and Ferrieri, 2022). Thus, mNGS has great potential because it provides rapid and reliable identification of etiological pathogens, and allows clinicians to select the most appropriate treatment regimens for patients who have complex MIs.

We conducted a single-center retrospective study to investigate the value of mNGS for the diagnosis of NTM in patients with MIs. We described the details of this process for 36 patients with MIs who presented with atypical clinical symptoms and lung imaging features and who had poor resolution of symptoms following empirical treatment or treatment based on sputum culture results. We ultimately confirmed the presence of a MI with an NTM in each patient. This study aims to improve understanding of the value of mNGS for the diagnosis of NTM in patients with MIs.

## Methods

### Patients and sample collection

This study initially examined 36 patients who had complex MIs and received mNGS testing for detection of NTM at the Second Hospital of Jilin University (Changchun, China) between Nov 2023 and Jun 2024. Each included patient had poor outcome from empirical treatment before admission, agreed to undergo testing of bronchoalveolar lavage fluid with NGS, and had NGS results indicating MIs with NTM. Clinical data and basic information (age, sex, etc.) were collected from all patients. Data from laboratory tests and imaging, such as routine blood parameters and chemistry, erythrocyte sedimentation rate (ESR), C-reactive protein (CRP), procalcitonin (PCT), and chest computed tomography (CT), were also collected. All patients underwent bronchofiberscopy for collection of BALF, and 8 patients received transbronchial lung biopsy (TBLB). The mNGS procedures, including nucleic acid extraction, library construction, and shotgun sequencing, were performed using the Illumination NextSeq 550DX. Bioinformatics analysis was carried out by WillingMed Technology Co, Ltd. (Beijing, China). A sample with 10 or more qualified reads was considered to indicate a pathogenic species.

### NTM smear and culture

Acid-fast bacilli (AFB) were detected by Ziehl-Neelsen staining of smears prepared from either bronchoalveolar lavage fluid or concentrated 24-hour sputum samples. For NTM culture, we adopted the conventional Löwenstein-Jensen method.

### Library preparation and metagenomic sequencing

DNA library construction was performed by automated nucleic acid extraction, enzymatic fragmentation, end-repair, terminal adenylation, and adaptor ligation, as described previously (Luan et al., 2021). Finished libraries were quantified by real-time polymerase chain reaction(PCR) (KAPA HiFi Kit, Roche) and pooled. Shotgun sequencing was performed using the Illumina Nextseq platform. Approximately 20 million 50 bp single-end reads were generated for each library. Bioinformatic analysis was conducted as described previously (Shen et al., 2021). Briefly, sequences of human origin were filtered (GRCh38.p13), and the remaining reads were aligned to a reference database (NCBI nt, GenBank, and an in-house curated genomic database) to identify microbial species and read counts. For each sequencing run, a negative control (culture medium with 10^4^ Jurkat cells/mL) was included.

### mNGS reporting criteria

Microbial reads identified from a library were reported if: (*i*) the sequencing data passed the quality control filters (library concentration > 50 pM, Q20 > 85%, Q30 > 80%) and (*ii*) the negative control (NC) in the same sequencing run did not contain the species or an excess of reads per million (RPM[sample]/RPM[NC] ≥ 5). This criterion was determined empirically in previous studies as a cutoff for discriminating true-positives from background contamination (Schlaberg et al., 2017; Wilson et al., 2019; Luan et al., 2021).

### Ethics statement

This study was approved by the Ethics Committee of the Second Hospital of Jilin University (approval number: 2024-166). All patients gave written informed consent for the analysis of personal and clinical details, along with any identifying images, to be published in this study. All methods were performed in accordance with the relevant guidelines and regulations.

### Statistical analyses

SPSS (version 21.0, USA) was used for all statistical analyses. Normally distributed variables are expressed as means ± standard deviations (SDs) and categorical variables as counts and percentages.

## Results

### Clinicopathological data

The 36 patients had an age range of 38 to 83 years (mean: 62.4 years). The Body Mass Index(BMI) range was 11.72 to 31.25 kg/m^2^ (mean: 23.11 kg/m^2^), and there were 13 patients with BMI greater than 25 kg/m^2^ and 3 patients with BMI below 18.5 kg/m^2^ (**Table 1**). Seven patients had hypertension, 6 had a pulmonary malignancy, 6 had coronary heart disease, 4 had bronchiectasis, 4 had cerebrovascular disease, 1 had phthisis, and the remaining 12 patients had good general health with no underlying diseases. All patients had at least one respiratory symptom, such as fever, cough, production of sputum, and dyspnea. Cough was the most common symptom (25 cases, 69.4%), followed by fever and sputum production (22 cases, 61.1%).

**Table 1:** Characteristics of patients upon admission (n=36).

Characteristic	N (%)
Demographics	
Male sex	16 (44.4%)
Age (years)	62.4 ± 11.5
Height (m)	1.65 ± 0.07
Weight (kg)	62.4 ± 11.5
Underlying diseases	
Cerebrovascular disease	4 (11.1%)
Coronary heart disease	6 (16.7%)
Bronchiectasis	4 (11.1%)
Hypertension	7 (19.4%)
Phthisis	1 (2.8%)
Comorbidities (first diagnosis)	
Lung cancer	6 (16.7%)
No underlying disease	12 (33.3%)
Clinical symptoms	
Fever	22 (61.1%)
Cough	25 (69.4%)
Sputum production	22 (61.1%)
Dyspnea	19 (52.8%)
Laboratory indexes	
Positive sputum culture	0 (0%)
Positive sputum acid-fast staining	3 (8.3%)^a^
Pulmonary CT findings	
Interstitial pneumonia	6 (16.7%)
Atelectasis	3 (8.3%)
Consolidation	28 (77.8%)
Pleural effusion	10 (27.8%)
Bronchiectasis	4 (11.1%)

a Patients #2, #10, and #25.

### Laboratory data

Laboratory examinations showed that 19 patients had increased levels of CRP, and 6 had increased levels of the white blood cell count, neutrophil ratio and count, and procalcitonin. The ESR was increased in 81.8% (18/22) of tested patients. All 36 patients underwent fiberoptic bronchoscopy, but there were no abnormalities in the airways of 28 cases. There was no evidence of *Mycobacterium tuberculosis* or NTM in the sputum and BALF cultures of all 36 patients. Three patients tested positive in acid-fast staining of BALF (3/36, 8.3%).

**Figure 1:**
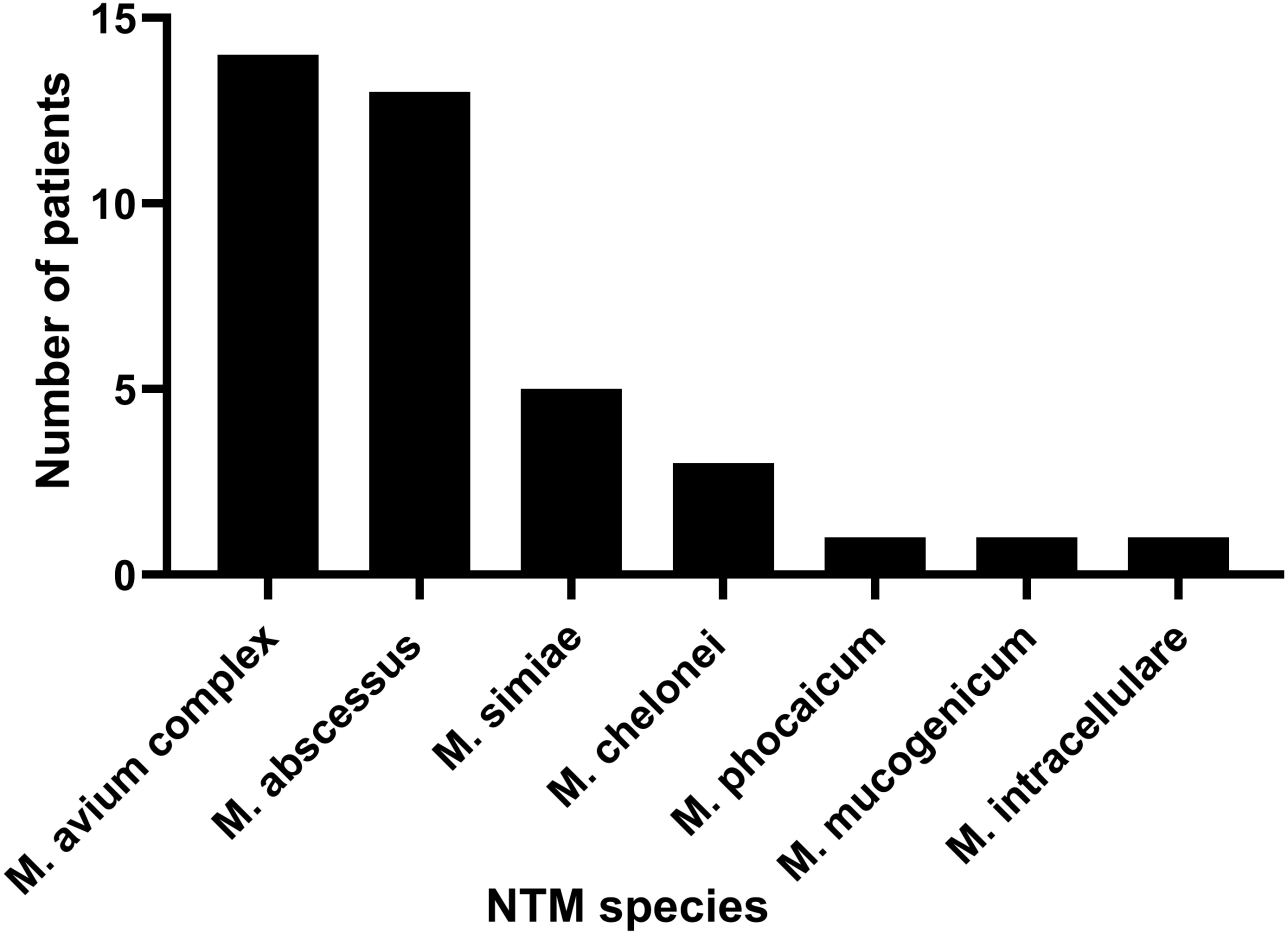
The number of patients with NTM strain infection based on the mNGS test results.

**Figure 2:**
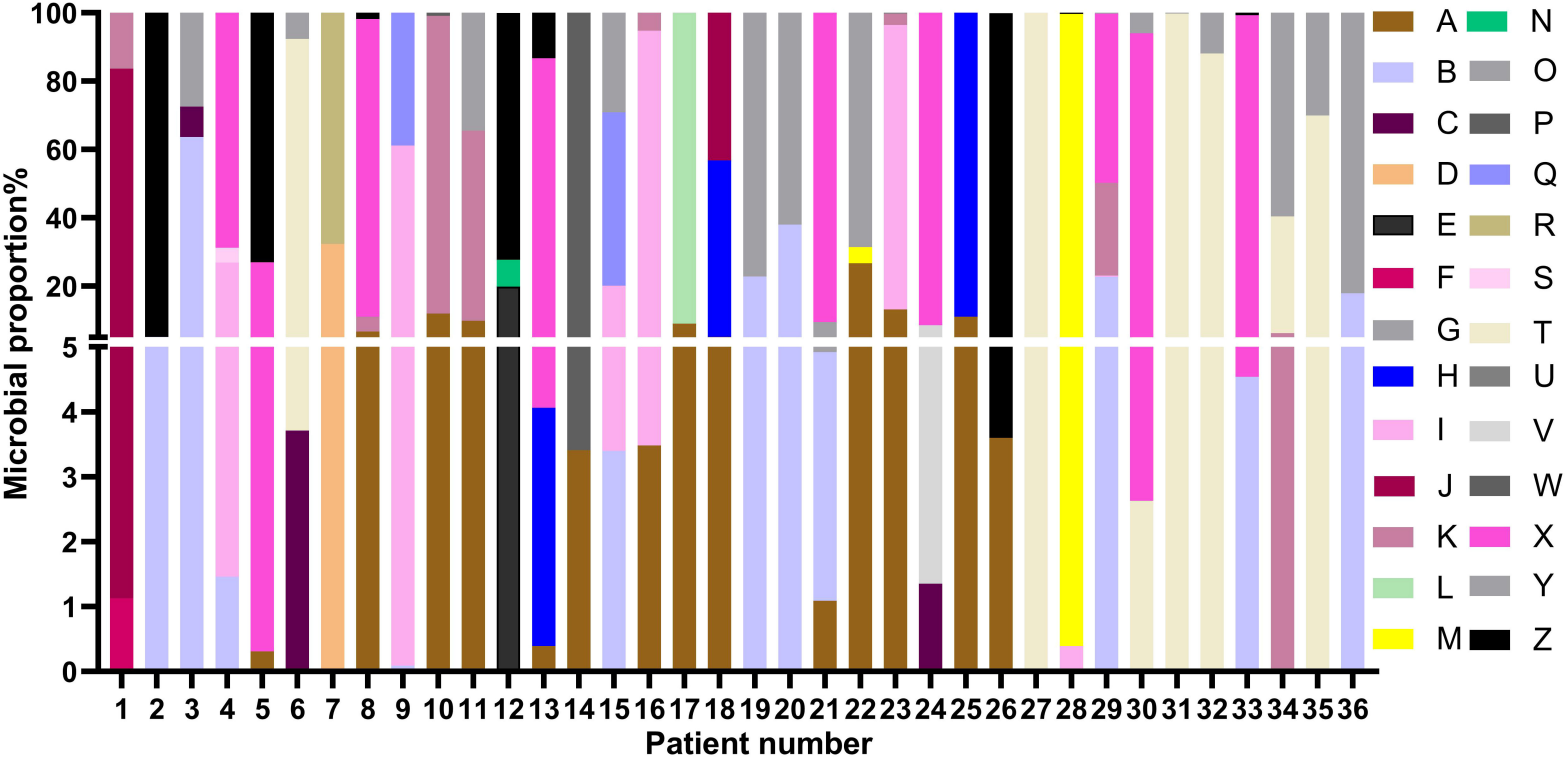
Relative abundances of NTM and non-NTM pathogens in each patient based on mNGS of BALF. The colors represent different pathogenic microorganisms (from A to Z) and length represents the percentage of pathogenic microorganisms. **(A)**, *M. avium* complex; **(B)**, *M. abscessus*; **(C)**, *M. chelonae*; **(D)**, *M. phocaicum*; **(E)**, *M. mucogenicum*; **(F)**, *M. intracellulare*; **(G)**, *M. simiae*; **(H)**, *Haemophilus parainfluenzae*; **(I)**, *Candida*; **(J)**, *Neisseria sicca*; **(K)**, *Pseudomonas aeruginosa*; **(L)**, *Burkholderia cepacia*; **(M)**, *Pseudomonas maltophilia*; **(N)**, *Staphylococcus aureus*; **(O)**, *Corynebacterium accolens*; **(P)**, *Nocardia farcinica*; **(Q)**, *Enterococcus faecium*; **(R)**, *Staphylococcus saprophyticus*; **(S)**, *Elizabethkingia anophelis*; **(T)**, Viridans streptococci; **(U)**, *Pneumocystis jiroveci*; **(V)**, *Trichoderma harzianum*; **(W)**, *Acinetobacter baumannii*; **(X)**, Virues; **(Y)**, *Aspergillus;* **(Z)***, Klebsiella pneumoniae*.

All patients had clear evidence pulmonary infections but poor response to empirical therapy, leading to the use of mNGS of BALF. The mNGS results identified NTM in all 36 patients (**Figure 1**; **Table 2**). These 7 NTM species were *M. avium complex* (14 patients), *M. abscessus* (13 patients), *M. simiae* (5 patients), *M. chelonae* (3 patients), *M. phocaicum* (1 patient), *M. mucogenicum* (1 patients), and *M. intracellulare* (1 patient). All patients were also complicated with infections by other bacteria, fungi, or viruses (**Figure 2**; **Tables 2**, **3**). The most common co-occurring species were *Pseudomonas aeruginosa* (8, 22.2%), *Klebsiella pneumoniae* (8, 22.2%), and *Aspergillus* (11, 30.6%). The first mNGS result in Patient #7 was negative, but acid smear of the BALF was positive, and our re-examination using mNGS combined with qPCR confirmed *M. phocaicum*.

**Table 2:** Underlying diseases, relevant history, and mNGS results of each patient.

Patient	Underlying disease and relevant history	mNGS results			
		NTM	Bacteria	Fungi	Viruses
#1	CHD, CVD, CA	M. intracellulare	Neisseria sicca, Pseudomonas aeruginosa	None	None
#2	CA	M. abscessus	Klebsiella pneumoniae	None	None
#3	Bronchiectasis, COPD, DM	M. abscessus, M. chelonae	None	Aspergillus	None
#4	ILD, CHD	M. abscessus	Elizabethkingia anophelis	Candida	Epstein-Barr virus
#5	Bronchiectasis, CVD	M. avium complex	Klebsiella pneumoniae	None	Rhinovirus
#6	DM	M. chelonae	Viridans Streptococci	Aspergillus	None
#7	None	M. phocaicum	Staph. Saprophytics	None	None
#8	Bronchiectasis	M. avium complex	Pseudomonas aeruginosa, Klebsiella pneumoniae	None	Adenovirus
#9	Bronchiectasis	M. abscessus	Enterococcus Faecium	Candida	None
#10	Bronchiectasis, PTB, CB	M. avium complex	Pseudomonas aeruginosa, Acinetobacter baumannii	None	None
#11	DM	M. avium complex	Pseudomonas aeruginosa	Aspergillus	None
#12	None	M. mucogenicum	Klebsiella pneumoniae, Staphylococcus aureus	None	None
#13	None	M. avium complex	Klebsiella pneumoniae, Haemophilus parainfluenzae,	None	Adenovirus
#14	None	M. avium complex	Nocardia farcinica	None	None
#15	HTN, CVD	M. abscessus	Enterococcus Faecium	Candida, Aspergillus niger	None
#16	HTN, CA	M. avium complex	Haemophilus parainfluenzae, Pseudomonas aeruginosa	None	None
#17	HTN, CVD	M. avium complex	Burkholderia cepacia	None	None
#18	None	M. avium complex	Haemophilus parainfluenzae, Neisseria sicca	None	None
#19	DM	M. abscessus	None	Aspergillus fumigatus	None
#20	COPD, HTN	M. abscessus	Corynebacterium accolens	None	None
#21	COPD, HTN	M. avium complex, M. abscessus	Corynebacterium accolens	None	Epstein-Barr virus
#22	None	M. avium complex	Pseudomonas maltophilia	Aspergillus sydowii	None
#23	Raising pigeons	M. avium complex	Pseudomonas aeruginosa	Pneumocystis jiroveci, Candida	None
#24	None	M. chelonae	None	Trichoderma harzianum	SARS-CoV2
#25	Contact with decaying plants	M. avium complex	Haemophilus parainfluenzae	None	None
#26	None	M. avium complex	Klebsiella pneumoniae	Aspergillus	None
#27	ILD, Lung cancer	M. abscessus	Viridans Streptococci	None	None
#28	Lung cancer	M. abscessus	Pseudomonas maltophilia, Klebsiella pneumoniae	Candida	None
#29	Vasculitis	M. abscessus	Pseudomonas aeruginosa	Candida, Aspergillus	Influenza A virus
#30	CHD	M. simiae	Viridans Streptococci	None	Influenza A virus
#31	HTN, CHD	M. simiae	Viridans Streptococci	Aspergillus, Pneumocystis jiroveci	None
#32	None	M. simiae	Viridans Streptococci	None	None
#33	HTN	M. abscessus	Klebsiella pneumoniae	None	Influenza A virus
#34	Lung cancer	M. simiae	Viridans Streptococci, Pseudomonas aeruginosa	Aspergillus	None
#35	None	M. simiae	Viridans Streptococci	Aspergillus	None
#36	None	M. abscessus	None	Aspergillus	None

CHD, coronary Heart Disease; CVD, cerebrovascular disease; CA, cancer (Non lung cancer); COPD, chronic obstructive pulmonary disease; DM, diabetes; ILD, Interstitial lung disease; HTN, hypertension; PTB, pulmonary tuberculosis; CB, chronic bronchitis; SARS-CoV2, Severe Acute Respiratory Syndrome Coronavirus 2.

**Table 3:** Sequence number of NTM and non-NTM pathogens in each patient based on mNGS of BALF.

Patient	A	B	C	D	E	F	G	H	I	J	K	L	M	N	O	P	Q	R	S	T	U	V	W	X	Y	Z
#1	0	0	0	0	0	1000	0	0	0	73214	14459	0	0	0	0	0	0	0	0	0	0	0	0	0	0	0
#2	0	920	0	0	0	0	0	0	0	0	0	0	0	0	0	0	0	0	0	0	0	0	0	0	0	16838
#3	0	447	62	0	0	0	0	0	0	0	0	0	0	0	0	0	0	0	0	0	0	0	0	0	193	0
#4	0	340	0	0	0	0	0	0	5941	0	0	0	0	0	0	0	0	0	1002	0	0	0	0	16034	0	0
#5	100	0	0	0	0	0	0	0	0	0	0	0	0	0	0	0	0	0	0	0	0	0	0	8701	0	23789
#6	0	0	150	0	0	0	0	0	0	0	0	0	0	0	0	0	0	0	0	3587	0	0	0	0	309	0
#7	0	0	0	11	0	0	0	0	0	0	0	0	0	0	0	0	0	23	0	0	0	0	0	0	0	0
#8	770	0	0	0	0	0	0	0	0	0	509	0	0	0	0	0	0	0	0	0	0	0	0	11532	0	203
#9	0	618	0	0	0	0	0	0	407431	0	0	0	0	0	0	0	259144	0	0	0	0	0	0	0	0	0
#10	730	0	0	0	0	0	0	0	0	0	5329	0	0	0	0	0	0	0	0	0	0	0	52	0	0	0
#11	88	0	0	0	0	0	0	0	0	0	498	0	0	0	0	0	0	0	0	0	0	0	0	0	309	0
#12	0	0	0	0	68	0	0	0	0	0	0	0	0	27	0	0	0	0	0	0	0	0	0	0	0	247
#13	157	0	0	0	0	0	0	1490	0	0	0	0	0	0	0	0	0	0	0	0	0	0	0	33590	0	5402
#14	373	0	0	0	0	0	0	0	0	0	0	0	0	0	0	10555	0	0	0	0	0	0	0	0	0	0
#15	0	14	0	0	0	0	0	0	69	0	0	0	0	0	0	0	209	0	0	0	0	0	0	0	120	0
#16	358	0	0	0	0	0	0	0	9387	0	539	0	0	0	0	0	0	0	0	0	0	0	0	0	0	0
#17	71	0	0	0	0	0	0	0	0	0	0	717	0	0	0	0	0	0	0	0	0	0	0	0	0	0
#18	79	0	0	0	0	0	0	795	0	664	0	0	0	0	0	0	0	0	0	0	0	0	0	0	0	0
#19	0	16	0	0	0	0	0	0	0	0	0	0	0	0	0	0	0	0	0	0	0	0	0	0	54	0
#20	0	116	0	0	0	0	0	0	0	0	0	0	0	0	189	0	0	0	0	0	0	0	0	0	0	0
#21	14	49	0	0	0	0	0	0	0	0	0	0	0	0	58	0	0	0	0	0	0	0	0	1159	0	0
#22	187	0	0	0	0	0	0	0	0	0	0	0	33	0	0	0	0	0	0	0	0	0	0	0	479	0
#23	26493	0	0	0	0	0	0	0	167650	0	6605	0	0	0	0	0	0	0	0	0	496	0	0	0	0	0
#24	0	0	148	0	0	0	0	0	0	0	0	0	0	0	0	0	0	0	0	0	0	794	0	10054	0	0
#25	115	0	0	0	0	0	0	927	0	0	0	0	0	0	0	0	0	0	0	0	0	0	0	0	0	0
#26	66208	0	0	0	0	0	0	0	0	0	0	0	0	0	0	0	0	0	0	0	0	0	0	0	2986	1768132
#27	0	30	0	0	0	0	0	0	0	0	0	0	0	0	0	0	0	0	0	134222	0	0	0	0	0	0
#28	0	66	0	0	0	0	0	0	907	0	0	0	249787	0	0	0	0	0	0	0	0	0	0	0	0	886
#29	0	109	0	0	0	0	0	0	2	0	130	0	0	0	0	0	0	0	0	0	0	0	0	238	1	0
#30	0	0	0	0	0	0	390	0	0	0	0	0	0	0	0	0	0	0	0	172	0	0	0	5973	0	0
#31	0	0	0	0	0	0	348	0	0	0	0	0	0	0	0	0	0	0	0	139374	0	0	0	0	0	0
#32	0	0	0	0	0	0	274	0	0	0	0	0	0	0	0	0	0	0	0	2026	0	0	0	0	0	0
#33	0	117	0	0	0	0	0	0	0	0	0	0	0	0	0	0	0	0	0	0	0	0	0	2445	0	17
#34	0	0	0	0	0	0	113	0	0	0	12	0	0	0	0	0	0	0	0	66	0	0	0	0	2	0
#35	0	0	0	0	0	0	129	0	0	0	0	0	0	0	0	0	0	0	0	310	0	0	0	0	4	0
#36	0	153	0	0	0	0	0	0	0	0	0	0	0	0	0	0	0	0	0	0	0	0	0	0	699	0

A, M. avium complex; B, M. abscessus; C, M. chelonae; D, M. phocaicum; E, M. mucogenicum; F, M. intracellulare; G, M. simiae; H, Haemophilus parainfluenzae; I, Candida; J, Neisseria sicca; K, Pseudomonas aeruginosa; L, Burkholderia cepacia; M, Pseudomonas maltophilia; N, Staphylococcus aureus; O, Corynebacterium accolens; P, Nocardia farcinica; Q, Enterococcus faecium; R, Staphylococcus saprophyticus; S, Elizabethkingia anophelis; T, Viridans streptococci; U, Pneumocystis jiroveci; V, Trichoderma harzianum; W, Acinetobacter baumannii; X, Virues; Y, Aspergillus; Z, Klebsiella pneumoniae.

### Imaging results

**Figure 3:**
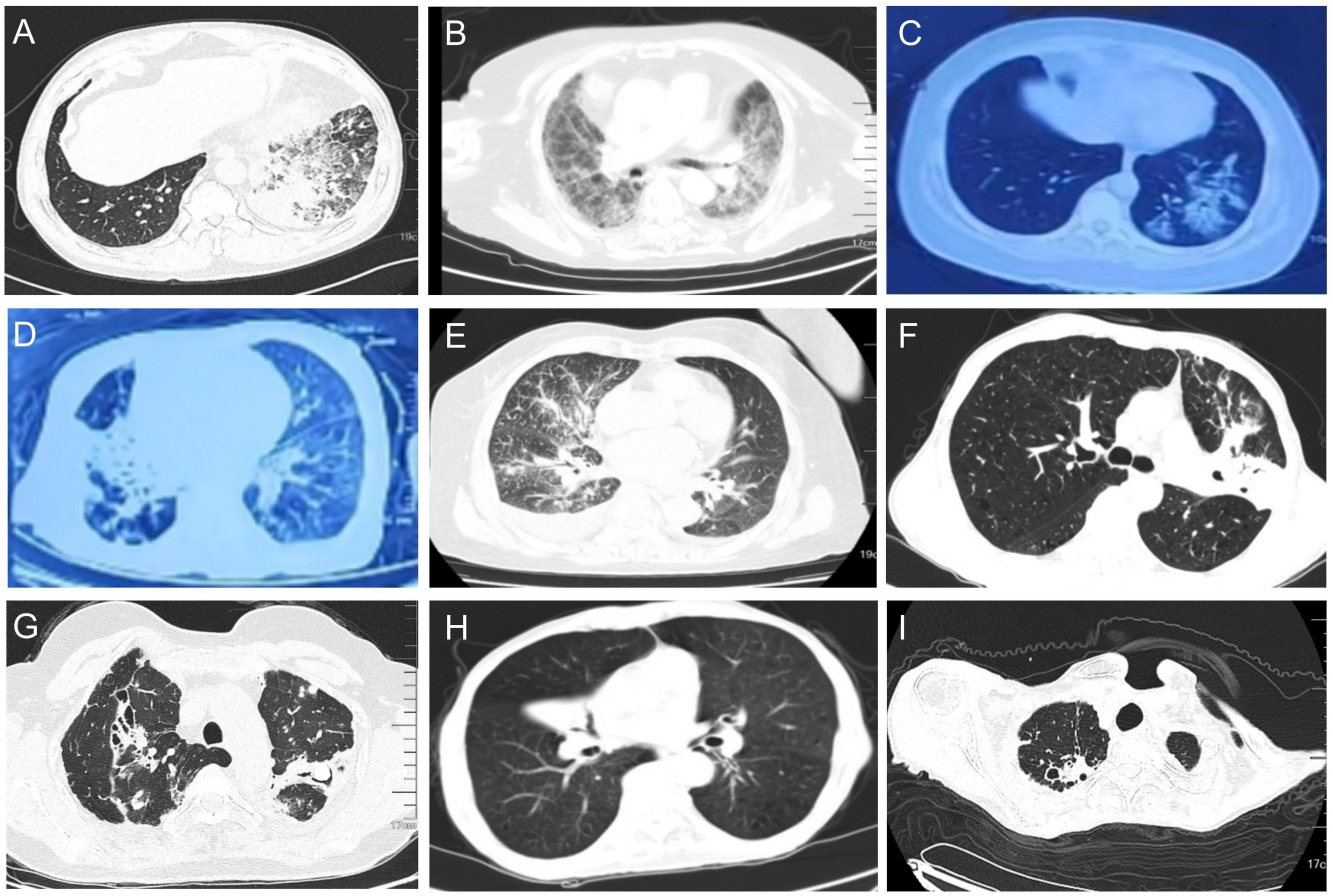
Pulmonary CT (lung window) representative findings of NTM patients at admission. **(A)** Consolidation accompanied with ground-glass opacity were observed in the lower lobe of the left lung. Widened interlobular septa were also observed. **(B)** Diffuse interstitial changed in the lungs. **(C)** Ground glass opacities in the lower lobe of the left lung. **(D)** Consolidation of the middle lobe of the right lung were observed, with associated traction bronchiectasis within the consolidated lesion. **(E)** The right bronchovascular bundle thickened, with multiple nodular diffusely distributed in the lung fields. A small amount of right pleural effusion presented. **(F)** Consolidation in the left upper lobe with cavitation, accompanied by surrounding fibrous strands and patchy opacities. **(G)** Consolidation in the upper lobes with traction bronchiectasis. A cavitary lesion is noted within the consolidation of the left lobe, containing an intracavitary spherical opacity. Scattered micronodules presented in the surrounding parenchyma. **(H)** Right middle lobe consolidation with bronchial cutoff sign and intralesional bronchiectasis. **(I)** Consolidation with cavitation in the right upper lobe, accompanied by surrounding fibrotic strands.

On admission, all 36 patients received chest CT examinations. The imaging findings had a variety of pathological patterns, such as consolidation accompanied with ground-glass opacity, widened interlobular septa, diffuse interstitial changed in the lungs (**Figures 3A–C**), consolidation with associated traction bronchiectasis (**Figure 3D**), bronchovascular bundle thickened, with multiple nodular diffusely distributed in the lung fields and pleural effusion presented (**Figure 3E**), consolidation with cavitation, and accompanied by surrounding fibrous strands and patchy opacities (**Figure 3F**), cavitary lesion containing an intracavitary spherical opacity (**Figure 3G**), consolidation with bronchial cutoff sign and bronchiectasis (**Figure 3H**), consolidation with cavitation in the right upper lobe, accompanied by surrounding fibrotic strands (**Figure 3I**).

### Co-infections

The co-infections were mainly by Gram-positive cocci and Gram-positive bacilli. Among all 36 patients, 32 (88.9%) had infections by bacteria, 17 (47.2%) had infections by fungi, and 24 (66.7%) had an underlying disease with co-infection. A mixed infection can increase the difficulty of treatment and prolong the treatment time.

### Pathological outcome

**Figure 4:**
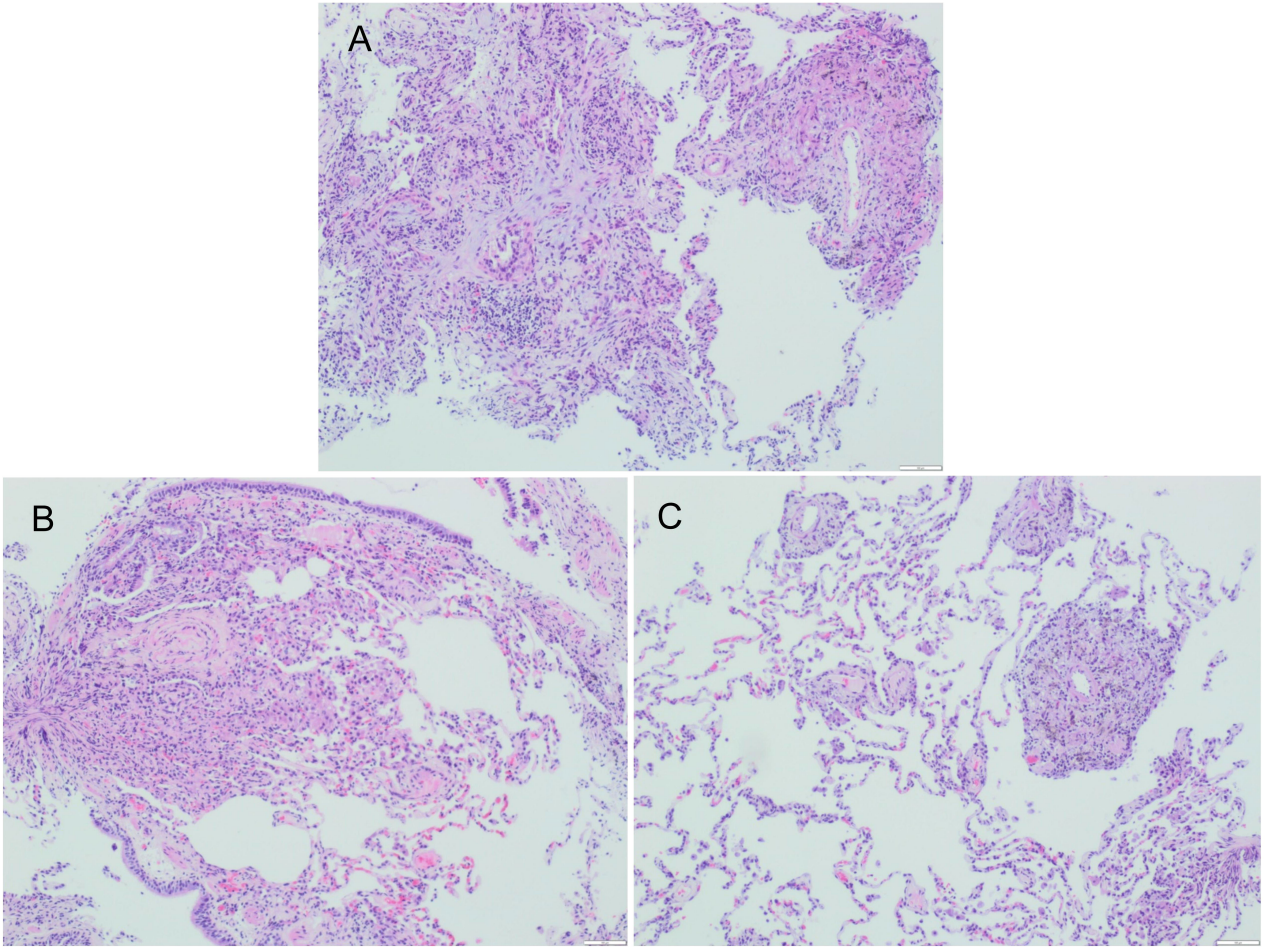
Fibrobronchoscopic lung biopsy specimen of Patient #2, **(A–C)** showing granuloma (H$E staining).

14 patients received TBLB, and 5 of them had typical granulomas with necrosis (**Figure 4**) with positive acid-fast staining. Eight cases had chronic inflammatory cell infiltration.

### Treatments and outcome

**Figure 5:**
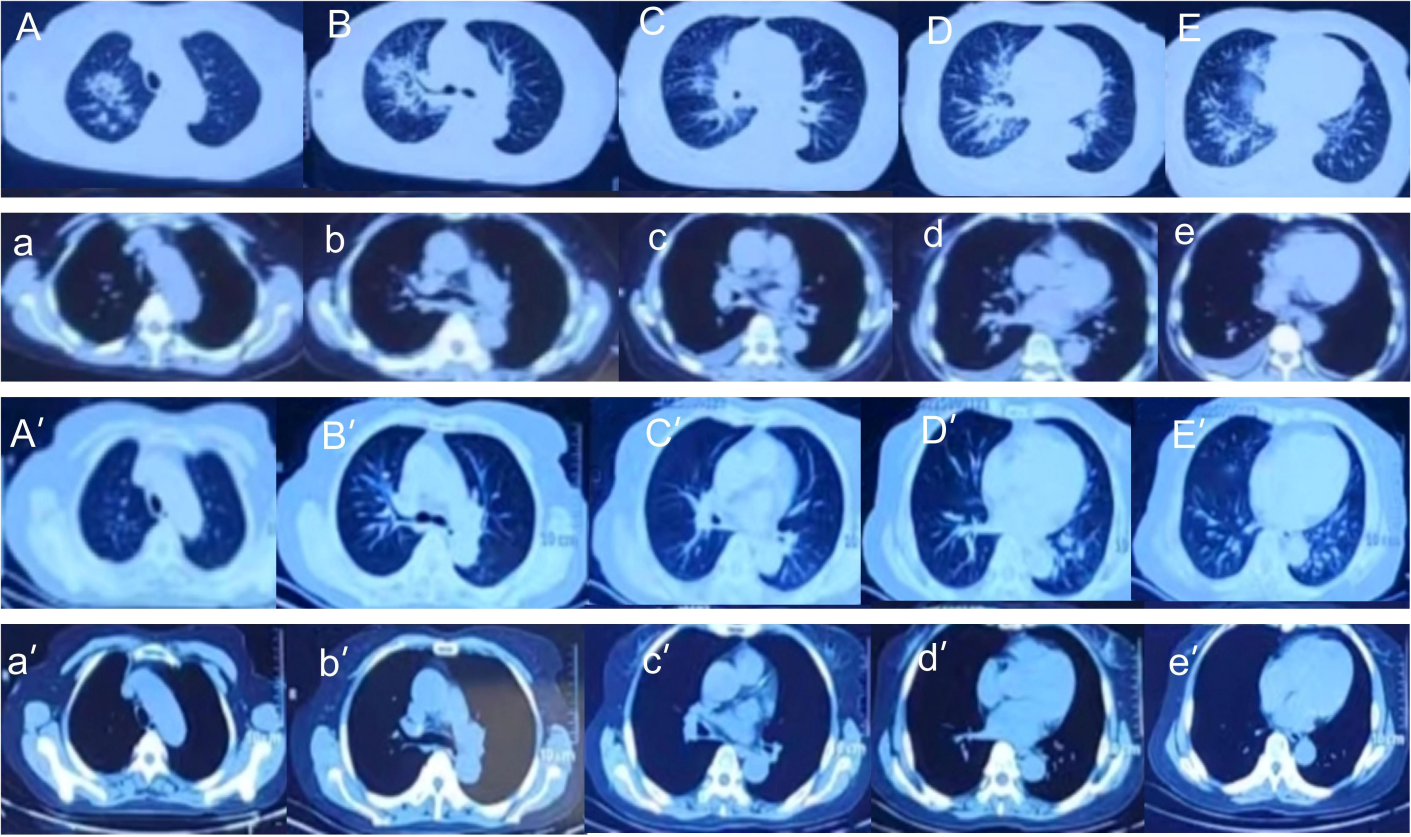
Chest CT imaging of Patient #1 before treatment (top 2 rows) and after treatment (bottom 2 rows). **(A–E, a-e)** Right upper lung consolidation with thickening of the bronchovascular bundle at the right lung. Scattered ground-glass opacities and nodulars were distributed throughout the pulmonary parenchyma. Right pleural effusion and enlarged right hilar lymph nodes were also observed. **(A’–E’, a ‘- e’)** Follow-up imaging demonstrated significant resolution of the right lung abnormalities. The previously noted right pleural effusion has completely resolved, and the right hilar lymph nodes showed reduction in size.

Immediately after admission, all patients received empirical anti-infection therapy with a beta-lactamase inhibitor or carbapenem or quinolone, and some patients received anti-fungal treatment. However, these treatments did not lead to significant relief of clinical symptoms in any of the 36 patients, and the pulmonary CT lesions were not absorbed in re-examination. This led to the use of mNGS of BALF. The results from mNGS indicated that all patients had infections by NTM, along with other bacteria (*Klebsiella pneumoniae*, *Staphylococcus saprophyticus*, *Enterococcus faecium*, *Burkholderia cenocepacia*, *Haemophilus parainfluenzae*, *Neisseria sicca*), fungi (*Candida*, *Aspergillus*, *Pneumocystis yersoni*), or viruses (Adenovirus, Rhinovirus, Epstein-Barr virus, Severe Acute Respiratory Syndrome Coronavirus 2(SARS-CoV2), Influenza A virus). After the NTM diagnoses, the anti-bacteria regimens were adjusted by adding different appropriate agents (Rifamycin, Ethambutol, Roxithromycin) according to guidelines for diagnosis and treatment of nontuberculous mycobacteriosis (**Table 4**) (Chinese Medical Association Tuberculosis Branch, 2020). At the same time, the treatments with other anti-infective or antifungal drugs were also adjusted(oseltamivir, fluconazole, or voriconazole) based on the mNGS results. The clinical manifestations in all 36 patients gradually resolved after treatment with roxithromycin, rifampicin, ethambutol, which lasted for 12 months. **Figure 5** shows the chest CT results of a typical patient before and after NTM treatment. Chest CT (**Figures 5A–E, a-e**) showed right upper lung consolidation with thickening of the bronchovascular bundle at the right lung. Scattered ground-glass opacities and nodulars were distributed throughout the pulmonary parenchyma. Right pleural effusion and enlarged right hilar lymph nodes were also observed. (**Figures 5A’–E’, a ‘- e’**) Follow-up imaging demonstrated significant resolution of the right lung abnormalities. The previously noted right pleural effusion has completely resolved, and the right hilar lymph nodes showed reduction in size.

**Table 4:** Antibiotics used before and after mNGS, and outcome of each patient.

Patient	Antibiotics Before mNGS	Clinical symptoms and lung CT	Antibiotics After mNGS	Clinical symptoms and lung CT
#1	Piperacillin	Not improved	Amikacin, Tigacycline, imipenem/Cilastatin, Clarithromycin	Improved
#2	Meropenem	Not improved	Amikacin, Tigacycline, imipenem/Cilastatin, Clarithromycin	Improved
#3	Meropenem, Fluconazole	Not improved	Amikacin, Tigacycline, imipenem/Cilastatin, Clarithromycin,Voriconazole	Improved
#4	Moxifloxacin	Not improved	Amikacin, Tigacycline, imipenem/Cilastatin, Clarithromycin, fluconazole	Improved
#5	Meropenem	Not improved	Amikacin, Tigacycline, imipenem/Cilastatin, Clarithromycin	Improved
#6	Piperacillin, Voriconazole	Not improved	Amikacin, Tigacycline, imipenem/Cilastatin, Clarithromycin,Voriconazole	Improved
#7	Meropenem	Not improved	Amikacin, Tigacycline, imipenem/Cilastatin, Clarithromycin	Improved
#8	Meropenem	Not improved	Amikacin, Tigacycline, imipenem/Cilastatin, Clarithromycin	Improved
#9	Meropenem	Not improved	Amikacin, Tigacycline, imipenem/Cilastatin, Clarithromycin,Fluconazole	Improved
#10	Meropenem	Not improved	Amikacin, Tigacycline, imipenem/Cilastatin, Clarithromycin	Improved
#11	Piperacillin sodium, Fluconazole	Not improved	Amikacin, Tigacycline, imipenem/Cilastatin, Clarithromycin,Voriconazole	Improved
#12	Piperacillin	Not improved	Amikacin, Tigacycline, imipenem/Cilastatin, Clarithromycin	Improved
#13	Meropenem	Not improved	Amikacin, Tigacycline, imipenem/Cilastatin, Clarithromycin,	Improved
#14	Piperacillin	Not improved	Amikacin, Tigacycline, imipenem/Cilastatin, Clarithromycin,TMP-SMX	Improved
#15	Moxifloxacin	Not improved	Amikacin, Tigacycline, imipenem/Cilastatin, Clarithromycin, Cefoperazone,Voriconazole	Improved
#16	Piperacillin	Not improved	Amikacin, Tigacycline, imipenem/Cilastatin, Clarithromycin	Improved
#17	Piperacillin	Not improved	Amikacin, Tigacycline, imipenem/Cilastatin, Clarithromycin	Improved
#18	Cefazolin	Not improved	Amikacin, Tigacycline, imipenem/Cilastatin, Clarithromycin	Improved
#19	Levofloxacin, Fluconazole	Not improved	Amikacin, Tigacycline, imipenem/Cilastatin, Clarithromycin,Voriconazole	Improved
#20	Cefoperazone	Not improved	Amikacin, Tigacycline, imipenem/Cilastatin, Clarithromycin	Improved
#21	Cefoperazone	Not improved	Amikacin, Tigacycline, imipenem/Cilastatin, Clarithromycin,	Improved
#22	Piperacillin	Not improved	Amikacin, Tigacycline, imipenem/Cilastatin, Clarithromycin,Voriconazole	Improved
#23	Meropenem	Not improved	Amikacin, Tigacycline, imipenem/Cilastatin, Clarithromycin,TMP-SMX, Fluconazole	Improved
#24	Levofloxacin	Not improved	Amikacin, Tigacycline, imipenem/Cilastatin, clarithromycin, Amphotericin B, Molnupiravir	Improved
#25	Ceftriaxone	Not improved	Amikacin, Tigacycline, imipenem/Cilastatin, Clarithromycin	Improved
#26	Piperacillin	Not improved	Amikacin, Tigacycline, imipenem/Cilastatin, Clarithromycin, Voriconazole	Improved
#27	Piperacillin	Not improved	Amikacin, Tigacycline, imipenem/Cilastatin, Clarithromycin	Improved
#28	Levofloxacin	Not improved	Amikacin, Tigacycline, imipenem/Cilastatin, Clarithromycin, Fluconazole	Improved
#29	Ceftazidime, Fluconazole	Not improved	Amikacin, Tigacycline, imipenem/Cilastatin, Clarithromycin, Voriconazole, 0seltamivir	Improved
#30	Ceftazidime,	Not improved	Amikacin, Tigacycline, imipenem/Cilastatin, Clarithromycin, Oseltamivir	Improved
#31	Piperacillin	Not improved	Amikacin, Tigacycline, imipenem/Cilastatin, Clarithromycin, Voriconazole, TMP-SMX	Improved
#32	Moxifloxacin	Not improved	Amikacin, Tigacycline, imipenem/Cilastatin, Clarithromycin	Improved
#33	Ceftazidime	Not improved	Amikacin, Tigacycline, imipenem/Cilastatin, Clarithromycin, Oseltamivir	Improved
#34	Moxifloxacin, Levofloxacin	Not improved	Amikacin, Tigacycline, imipenem/Cilastatin, Clarithromycin, Voriconazole	Improved
#35	Piperacillin	Not improved	Amikacin, Tigacycline, imipenem/Cilastatin, Clarithromycin	Improved
#36	Levofloxacin	Not improved	Amikacin, Tigacycline, imipenem/Cilastatin, Clarithromycin, Voriconazole	Improved

## Discussion

The prevalence of NTM lung disease continues to increase globally, and it is now a widespread serious public health problem (Ahmed et al., 2020; Johansen et al., 2020; Varghese and Al-Hajoj, 2020). NTM pulmonary disease is a systemic disease caused by different species of NTM that mainly infect the lungs. The different species of NTM are common in natural environments, such as soil, dust, and water, and NTM infections are generally opportunistic and acquired from the environment. Although NTM species are generally less pathogenic to humans than *M. tuberculosis*, a human host with impaired local or systemic immune response can develop lung lesions. Most NTM lung diseases have slow onset, lack specific clinical manifestations, and are difficult to distinguish from other diseases (Cowman et al., 2019; Loret and Dumoutier, 2019; Chin et al., 2020; Sharma and Upadhyay, 2020; Nogueira et al., 2021). Clinicians may therefore ignore or overlook the existence of NTM lung disease, and patients are often misdiagnosed or remain undiagnosed.

### Clinical characteristics and laboratory examinations of NTM mixed infection

The clinical symptoms of the 36 patients in our study were variable, and included fever, cough, sputum production, and dyspnea (**Table 1**). The lung CT findings were also variable, with evidence of consolidation, nodules, pleural effusion, interstitial changes, and other abnormalities (**Table 1**, **Figure 3**). Most of our patients received anti-infection treatments at different hospitals before admission to our hospital, and their clinical symptoms and lung CT results showed no changes following these initial treatments. Due to the receipt of anti-microbial treatment before admission, some patients also had atypical symptoms and laboratory results. After admission to our hospital, we provided empirical antibacterial treatment and some patients also received empirical antifungal treatment. Because the clinical symptoms and lung CT results of the 36 patients did not change after this initial treatment, we performed mNGS detection of BALF in all 36 patients, and TBLB examination for 14 patients.

It is important to improve the understanding of NTM pulmonary disease and to identify NTM species as soon as possible so that prompt and appropriate treatment can be used. The traditional diagnosis of NTM infection relies on clinical manifestations, histopathology, acid-fast smears, and isolation of NTM from culture (Hahn et al., 1987; Maboni et al., 2024). In recent years, the T-SPOT^®^ test, qPCR, and other methods have also been used for detection and diagnosis of NTM infections (Ying et al., 2024). However, these methods can have low sensitivity, are time-consuming, and require special equipment, so many hospitals lack the capability to diagnose NTM pulmonary disease.

Our results are somewhat inconsistent with those from previous studies. Our sputum or BAL cultures were negative for NTM, and the positive rate from acid-fast staining was only 8.3% (3/36), significantly lower than that of mNGS. Moreover, positive acid-fast staining cannot distinguish tuberculosis(TB) from NTM, and use of these results alone could lead to misdiagnosis and inappropriate treatment (Xu et al., 2022). This may be because of the interference or dominance of other strains in the MIs, the small sample size, and possible sample bias. Our pathological positivity rate from TBLB was only 35.7%, possibly due to the small number of samples obtained from the TBLB. We also found that T-SPOT^®^ test for NTM had low sensitivity and specificity, consistent with the literature (Xu et al., 2022).

### Use of mNGS for the diagnosis of NTM mixed infections

mNGS can be used to confirm the presence of pathogenic microorganisms in the lower respiratory tract, and this method is also fast, highly accurate, low-cost, has wide coverage, and can be used to discover new pathogens (Zheng et al., 2021; Chen et al., 2022; Diao et al., 2022). mNGS can detect all nucleic acid fragments in a clinical sample, identify the species and types of different pathogens using bioinformatics analysis, and provide quantitative data (such as sequence number and coverage) of all pathogens. An advantage of mNGS is that it may lead to the administration of more appropriate antibiotics in patients with lower respiratory tract infections, and thereby decrease the risk of death (Yan et al., 2025). For example, mNGS can provide reliable diagnosis of invasive pulmonary aspergillosis (Yang et al., 2024) and detection of various pulmonary TB samples. A study of children with pneumonia showed that conventional microbial tests had a positivity rate of 68% relative to mNGS (100%) (Lu et al., 2024). mNGS is therefore an important tool for the etiological diagnosis of pulmonary TB and determining the microecological characteristics of the lungs (Zhou et al., 2024). A retrospective study that compared NTM detection rates from mNGS with the BACTEC Mycobacterial Growth Indicator Tube 960 (the current gold standard for diagnosis of NTM) showed that mNGS had greater sensitivity in analysis of BALF, lung needle biopsy specimens, and sputum (Wei et al., 2023). mNGS can detect NTM quickly and with high sensitivity in a variety of samples, and can also detect pathogens that occur as co-infections. In agreement with previous studies, we found that mNGS was reliable for the detection of mixed MIs and for identification of genes responsible for antibiotic resistance. An additional advantage of mNGS is that it can rapidly screen multiple drug resistance genes associated with the development of NTM disease, and thereby enable patients with multiple and extensively resistant bacterial infections to receive more effective and individualized treatment regimens (Liu et al., 2022; Xu et al., 2022; Matsumoto and Nakamura, 2023). Our retrospective analysis indicated that patients who had MIs with an NTM often had an underlying disease or a coexisting viral infection that contributed to immune dysfunction. The most common coexisting pathogens, including *P. aeruginosa*, *Aspergillus*, and several others, are associated with different coexisting underlying diseases, consistent with the traditional understanding of NTM as conditional pathogens (Hahn et al., 1987; Lopes et al., 2022).

### Limitations of NGS detection

The advancement of sequencing technology led to significant decreases in the cost of generating sequence data, but the total cost of required reagents and sample processing remains high. The cost of operating and maintaining the bioinformatics analysis process is also relatively high. The significantly higher total cost of mNGS compared to many other clinical methods greatly limits its use in routine clinical applications. However, with the standardization, optimization, and increased automation in mNGS, and the continuing increases in the possible application of mNGS in different clinical scenarios, the total cost of mNGS continues to decrease and it is increasingly accepted by more clinicians and patients (Liu et al., 2025; Qin et al., 2025).

Importantly, we used established auditing standards for NGS testing, and this allowed screening out of nonpathogenic (background) bacteria. At the same time, we also considered the patient’s medical history, clinical symptoms, physical signs, and imaging results when interpreting the NGS findings. After treatment, we observed the patient’s symptoms, laboratory results, and imaging results, especially when the NGS indicated evidence of NTM, which have lower pathogenicity than *M. tuberculosis*. This cautious approach to diagnosis is necessary because it can be difficult to clearly distinguish background bacteria from pathogenic bacteria. For example, empirical antibiotic treatment is usually ineffective in patients with chronic obstructive pulmonary disease (COPD) who experience difficulty breathing, coughing, and sputum production. However, if a pulmonary CT shows consolidation with a cavity shadow in the right middle lobe of the lung and the NGS result indicates drug-resistant *Klebsiella pneumoniae* and NTM, we will consider drug-resistant *Klebsiella pneumoniae* and NTM as pathogenic, rather than background. In this case, we adjust the anti-infective treatment according to the patient’s condition and provide anti-TB treatment. We also observe changes in the patient’s symptoms and imaging results to determine whether further adjustment of treatment is needed. Therefore, we did not rely solely on NGS results. Although NGS has high sensitivity, we still need to make a comprehensive judgment based on the patient’s medical history and other factors. At the same time, we also recognize the possible need to adjust the medications and observe changes in the patient’s condition when interpreting the NGS results.

In short, patients with NTM pulmonary infections can have diverse clinical symptoms and imaging findings, especially those with MIs, and this can lead to misdiagnosis. Therefore, the rapid and accurate identification of NTM and of co-occurring pathogens by mNGS has many important advantages, such as high efficiency, high sensitivity, and wide coverage. mNGS is particularly useful for the accurate identification of NTM.

This study had several limitations. First, it was a single-center retrospective analysis, and most samples were from hospitalized patients with who responded poorly to initial empirical treatment. Therefore, the sample size was small, and the rate of NTM detection was significantly higher than the reported prevalence rate of NTM in the general population. These factors could have biased our detection of NTM mixed bacterial infections. Future large and multi-center studies that use an appropriate control group should be used to analyze the morbidities and co-infection of patients admitted for NTM infection.

## Conclusions

As a new technology for etiological diagnosis, mNGS can provide improved sensitivity for the diagnosis of NTM pulmonary infections in patients who have MIs of the lungs. mNGS can also detect multiple bacteria, fungi, and viruses, and therefore reduce the incidence of misdiagnosis and delayed diagnosis and treatment. However, it is also necessary to consider diagnostic specificity and to use strict quality control measures when using mNGS for diagnosis of NTM pulmonary infections.

## Data Availability

The data presented in the study are deposited in the China National center for Bioinformation(CNCB) repository, accession number PRJCA041435.
